# Association between Hospital Nurses’ Perception of Patient Safety Management and Standard Precaution Adherence: A Cross-Sectional Study

**DOI:** 10.3390/ijerph16234744

**Published:** 2019-11-27

**Authors:** Ji-Hye Lim, Jung-Won Ahn, Youn-Jung Son

**Affiliations:** 1 Division of Nursing, Chung-Ang University Hospital, Seoul 06974, Korea; lo9190ve@naver.com; 2 Red Cross College of Nursing, Chung-Ang University, Seoul 06974, Korea; jwahn@cau.ac.kr

**Keywords:** hospital-acquired infection, nurses, patient safety management, standard precautions

## Abstract

Standard precautions should be applied to prevent health care-associated infections during every nursing activity. However, adherence to standard precautions was reported to be inadequate. Therefore, this study aimed to identify the rates of standard precaution adherence and the association between perception of patient safety management and standard precaution adherence. In this cross-sectional descriptive study, a convenience sample of nurses was recruited from a university-affiliated teaching hospital in Seoul, Korea. Data were collected using a structured self-report questionnaire. Among the 332 questionnaires returned (response rate: 94.9%), a total of 329 nurses were analyzed. In the present study, the overall standard precaution adherence rate was approximately 53.5%. The multiple linear regression results revealed that participants’ perceptions of patient safety management were only significantly associated with standard precaution adherence after adjusting other covariates (*β* = 0.412, *p* < 0.001). Nurse supervisors should focus more on raising awareness about nurses’ perception of patient safety management based on the specific work environment, such as the total number of nurses working together and the nurse-to-patient ratio. Nurse educators should develop integrated curricula to help graduate nurses transition smoothly into professional practice and enhance adherence to standard precautions in diverse health care settings.

## 1. Introduction

With the increase in the need for surgical or invasive procedures, emerging infectious diseases, and prevalence of multidrug-resistant bacteria, the role of health professionals in protecting patient safety has been emphasized [1]. Health care-associated infection can occur during a patient’s treatment process or many surgical procedures and poses a major threat to patient safety and care [2]. Health care-associated infections prolong hospital stays and increase the medical cost burden [3,4]. In Korea, the incidence of health care-associated infections has been reported to occur in 5% to 10% of hospitalized patients [5]. In 2015, 186 Korean cases of Middle East Respiratory Syndrome coronavirus (MERS-CoV) occurred, mostly due to inappropriate infection control within the hospital [6]. For these reasons, Korean health care authorities and the government have strongly emphasized the control and prevention of health care-associated infections [6,7]. In particular, standard precautions comprise the minimal infection prevention policy that must be followed regardless of the kind of infectious disease; however, several international studies have reported that health professionals’ adherence to standard precautions is suboptimal [1,3].

Patient safety consists of an absence of errors and preventable accidents that can occur during the health care process, and the elimination or alleviation of patient injury [8]. Patient safety management includes health care-associated infection prevention [2]. Globally, government engagement, and intervention in patient safety management in the health care environment is increasing [9]. In the United States, the Centre for Disease Control and Prevention and the Hospital Infection Control Practices Advisory Committee have recognized the negative impact of health care-associated infection on patients’ prognosis and the increased social costs due to increasing hospital infections. In 1996, they published a guideline for hospital isolation precautions; a revised version followed in 2007, and adherence among health care professionals was strongly recommended [10].

The Korean government adopted a Health Care Accreditation policy in 2004 to assess medical service quality and continuously strengthen insufficient areas [11]. Thus, the Patient Safety Act was enacted in early 2015. In 2017, the Korea Centers for Disease Control and Prevention and Korean Society for Health Care-associated Infection Control and Prevention developed a standardized health care-associated infection prevention guideline in accordance with the circumstances [5]. Adherence to standard precautions comprises the minimum infection prevention practices that apply to all patient care including hand hygiene, sharps safety, use of personal protection, respiratory hygiene; safe management of blood and body fluid spillages; and decontamination of equipment, waste, linen, and laundry [10]. While standard precautions serve as the universal infection prevention guidelines which must always be followed, the results of several studies focused on nurses have revealed low adherence rates [1,12].

Nurses interact closely with patients for 24 h and are the medical personnel with the highest contact frequency. Thus, nurses’ adherence to standard precautions in health care settings and awareness of patient safety management is essential to ensuring patient safety [13,14]. Few studies have explored factors such as awareness regarding the importance of patient safety management affecting standard precautions among hospital nurses in Korea. The standard precaution adherence rates of Korean nurses have been reported to range from 60% to 80% and vary according to the work environment. Importantly, while knowledge and awareness of standard precautions are high, the reported adherence rates were relatively low [15,16].

Before strategizing how to improve adherence rates, more studies are needed to identify the influential factors, including working conditions. Several studies have reported on individual factors such as knowledge, attitude burnout, clinical performance ability, experience, and participation in education [1,12,16,17,18,19]. At the organizational level, patient safety culture, personal protective equipment accessibility, and work environment [1,3,14] have been reported as factors that affect nurses’ adherence to standard precautions. Particularly, it is necessary that health professionals demonstrate a high level of commitment and a strong, cohesive spirit among various departments in health care settings [9,20]. Importantly, patient safety management should be maintained in all hospitals to provide better quality of care. In this context, nurses’ perception of patient safety management may be linked to workload demands, which involve lower safety activities such as infection control [21,22]. However, few studies have investigated whether nurses’ perceptions of patient safety management are related to their adherence to standard precautions.

This study aimed to describe hospital nurses’ adherence to standard precautions and identify the impact of their perception of patient safety management on adherence to standard precautions after adjusting for socio-demographic and work-related characteristics.

## 2. Methods 

### 2.1. Study Design and Setting

This descriptive cross-sectional study used a convenience sample of hospital nurses employed in a tertiary teaching, university-affiliated hospital in Seoul, Korea. The inclusion criteria were as follows: nurses with a minimum of one year of clinical experience in hospital settings. Nurses who did not participate in direct patient care were excluded. The number of subjects required for multiple linear regression analysis was calculated using the G*Power 3.1.9.2 program [23]. The sample size required for the multiple regression method was 292, with an effect size of 0.10, significance level of α = 0.05, power (1 − β) = 0.95, and 15 predictors. A self-report questionnaire was distributed to 350 nurses, considering the dropout rate of 20% [24]. Among the 332 questionnaires returned (response rate: 94.9%), 3 were excluded because the respondents had not included demographic characteristics (e.g., education level). Therefore, 329 questionnaires were analyzed. 

### 2.2. Measures

#### 2.2.1. Socio-Demographics and Work-Related Characteristics

Nurses’ socio-demographic characteristics consisted of age (years), marital status (married or partnered/single) and educational level (college/university/graduate school). The following work-related characteristics were measured: clinical experience (total years of practice, years of practice in current job), current position (staff/charge nurses), area of practice (medical ward/surgical ward/intensive care units/emergency department), number of nurses working together, nurse-to-patient ratio, working hours per week, prior training in patient safety (yes/no) and infection control education (yes/no).

#### 2.2.2. Adherence to Standard Precautions

Nurses’ adherence to standard precautions was assessed using the Compliance with Standard Precaution Scale (CSPS) [25]. Permission to use the CSPS was obtained from the original author via email. The tool was carried out in a forward–backward translation procedure. At first, two researchers, proficient in both English and Korean, independently translated the CSPS into Korean, and then a third researcher compared the two versions and selected the best translation. Next, the translated Korean CSPS was evaluated by ten nurse experts with more than 10 years of clinical experience and postgraduate degrees to verify understanding and cultural congruence as well as to evaluate content validity. The four attributes of the questions were rated on a 4-point scale (1 = “not relevant”; 4 = “highly relevant”). The content validity index was 0.87, with good congruence with the original version. To ensure the validity of the questionnaire, a pilot study was conducted two weeks prior to the main study. The questionnaire was distributed to 10 staff nurses to be tested for user-friendliness and clarity. No changes were made to the questionnaire after the pilot study as all questions were found to be clear. The questionnaire took 10 to 15 min to complete. Finally, the translation was sent to the original developer for another discussion to decide on the Korean translated version of the CSPS. The 20 items are rated on a 4-point Likert scale ranging from 1 to 4 (1 = “never”, 2 = “seldom”, 3 = “sometimes” and 4 = “always”). One point was given only to the response “always” and “never” to the reverse scoring item. No score was given to responses “seldom” and “sometimes”. The total scores ranged from 0 to 20, and a higher score signified higher adherence to standard precautions. For the original study [25], Cronbach’s α was 0.73; in this study, it was 0.82.

#### 2.2.3. Nurses’ Perception of Patient Safety Management

Nurses’ perception of patient safety management was assessed using the Perception of Importance on Patient Safety Management scale (PI-PSM), and permission to use it was obtained from the Korean original authors [22]. The 21 item questionnaire was categorized into four factors as “concern about patient safety management” (7 items), “confidence about patient safety management” (5 items), “will for patient safety management” (5 items), and “recognition about patient safety management” (4 items). Each item was rated on a 5-point Likert scale ranging from 1 to 5 (1 = “strongly disagree”, 2 = “disagree”, 3 = “neither”, 4 = “agree” and 5 = “strongly agree”). Higher scores indicated that recognition of the importance of patient safety management was high. The Cronbach’s α coefficient was 0.86 when the scale was developed and 0.93 in this study.

### 2.3. Statistical Analysis

Data were analyzed using the SPSS statistics version 23.0 (IBM Corp, Armonk, NY, USA) and tested at a significance level of 5% (two-sided). Descriptive statistics, which included frequencies, percentage, mean, and standard deviation, were used to report participants’ socio-demographic information and work-related characteristics. Differences in adherence to standard precautions by socio-demographics and work-related characteristics were analyzed using the independent *t*-test and one-way ANOVA. However, to investigate the difference in adherence to standard precautions by educational level, we used the Kruskal-Wallis test. Bonferroni adjustment was applied to multiple comparisons on continuous variables. The measurement tools’ internal reliability was tested using Cronbach’s α. Pearson, Spearman’s rho and Kendall’s tau correlation were performed. Multiple linear regression was conducted to identify associations among the main variables.

### 2.4. Ethical Considerations

Ethical approval was granted by the institutional review board (IRB No. 1732-001-274). The primary investigator contacted the hospital’s nurse managers and explained the aim of the study and data collection process. Data were collected from July to August in 2017. Two research assistants were then trained to assist with both the distribution and collection of the questionnaires in each of the participating hospital units. An information letter followed the questionnaire. Participants were informed about the study’s purpose and asked to provide their written consent.

## 3. Results

### 3.1. Nurses’ Socio-Demographics and Work-Related Characteristics

All the participants were female, the average age was 29.48 ± 4.50 years, and 242 (73.6%) were single. The majority were university graduates (73.9%) and staff nurses (83.0%). The average for the nurses’ total clinical experience was 6.74 ± 4.72 years. Participants were working in medical (31.3%), surgical (24.9%), or intensive care units (19.8%). The average nurse-to-patient ratio was one nurse to 10.25 ± 4.63 patients, and the average working hours per week were 44.57 ± 4.06. Most participants had previously attended patient safety education and infection control education course. The mean score of perception of patient safety management was 3.89 ± 0.46 (Table 1).

### 3.2. Nurses’ Adherence to Standard Precautions

Table 2 shows the five highest and lowest scored items for adherence to standard precautions. The average score of the standard precaution adherence was 10.71 ± 4.39, and the overall adherence rate was approximately 53.5%. The item with the highest adherence was “I put used sharp articles into sharps box” (94.8%), whereas the item with the lowest adherence was “I wear a gown or apron when exposed to blood, body fluids, or any patient excretions” (17.9%).

### 3.3. Differences in Adherence to Standard Precautions by Socio-Demographic and Work-Related Characteristics Among Nurses

Post hoc Bonferroni analysis showed significant differences in the total number of nurses working together, and the nurse-to-patient ratio. Namely, there was a higher adherence rate for the group with less than 20 nurses working together compared to the group with 20 to 39 nurses (*p* = 0.040). A nurse caring for 5 to 9 patients was more likely to adhere to standard precautions than a nurse looking after 15 or more patients (*p* = 0.046) (Table 3). 

### 3.4. Correlation between Measured Variables

Before conducting multiple linear regression analysis, we examined the correlation between the main variables (Table 4). Nurses’ perceptions of patient safety management were significantly positively correlated with adherence to standard precautions (*r* = 0.413, *p* < 0.001). On the other hand, there was a significant negative correlation between nurse-to-patient ratio and adherence to standard precautions (*rho* = −0.123, *p* = 0.026).

### 3.5. Influence of the Perceptions Regarding Patient Safety Management on Adherence to Standard Precautions

Before running a multiple linear regression, we identified which variables were affected by multicollinearity. The presence of multicollinearity was examined by a correlation matrix of all of the predictor variables and observing the variance inflation factor. The variance inflation factors were in the range 1.69–2.53. However, there was a correlation of 0.92 between age and total years in practice. Thus, the total years in practice was excluded in multiple linear regression analysis.

The multiple linear regression results showed that perception of patient safety management independently influenced nurses’ adherence to standard precautions (*β* = 0.412, *p* < 0.001, *R*^2^ change = 0.159) after adjusting for socio-demographic and work-related characteristics (Table 5).

## 4. Discussion

Nurses’ high level of commitment to ensuring proper infection control as an important part of patient safety management is pivotal to maintaining a safe patient environment [26]. Accordingly, nurses’ views on the importance of patient safety management are highly associated with adherence to standard precautions.

First, our study showed that the rates of adherence to standard precautions were found to be 10.71 points (out of 20), with an overall adherence rate of 53.5%. The standard precaution adherence rate found in this study was lower than the results of previous studies, namely, Cruz et al.’s findings [27], which showed that the average scores for standard precaution adherence were 12.93 points for nurses and 9.75 points for student nurses. According to Pereira et al.’s study [28], standard precaution adherence rates for Brazilian and Hong Kong nurses were approximately 69.4% and 57.4%, respectively. This discrepancy, compared to previous studies, could have been related to the shorter clinical experiences of the participants in this study. However, the standard precaution adherence rates in this study did not show a significant difference according to clinical experience. Diverse health care settings, including different working conditions or environments, may have also contributed to the inconsistency of this study’s results regarding adherence to standard precautions [1,14]. Thus, further studies should be conducted with a larger sample in diverse health care settings.

Next, the standard precaution adherence items with the two lowest adherence rates were (1) wearing personal protection equipment (such as a gown or apron), with an adherence rate of 17.9%, and (2) wearing a surgical mask alone or in combination with goggles, a face shield, and an apron, with a adherence rate of 32.2%. This finding was similar to the aforementioned study, which showed that Brazilian and Hong Kong nurses had low adherence rates for wearing personal protection equipment [28]. Further, our result supported some Korean studies which surveyed Korean hospital nurses [29,30], implying that, regardless of nurses’ cultural backgrounds, specialized training or education regarding the wearing of personal protective equipment must be well-executed. Suh and Oh [15] stated that the use of personal protective equipment hinders the formation of a therapeutic relationship with patients and decreases job efficiency due to the time consumed in taking it on it and off as well as the discomfort of wearing it. There were also physical difficulties, such as insufficient equipment and accessibility [1,29].

On the other hand, high-quality equipment and adequate communication systems can increase the “wearing rate” [4,31]. Thus, supportive training programs to raise nurses’ positive perceptions and attitudes are needed to improve their wearing of personal protective equipment. Particularly, nurse educators with organizational support should develop and provide specialized guidelines and interventions that identify the characteristics and problems unique to each department rather than a standardized approach for the entire nursing staff in a hospital.

In the present study, standard precaution adherence rates were relatively high in units with fewer than 20 nurses, such as newborn nurseries, delivery rooms, and psychiatric wards. Shin et al. [30] found that the standard precaution adherence rates of nurses in the newborn nurseries were higher than those of nurses in medical, surgical, and intensive care units. Since newborns are vulnerable to infections, and an environment with a large number of newborns is highly susceptible to the spread of infections, thorough implementation of infection prevention guidelines is important [32]. Such precautions reflect the specificity of departments that care for patients at high risk of infection, which likely result from safety management awareness and increased performance of safety precautions due to continuous infection rate monitoring and training. Additionally, the rate of adherence to standard precautions in this study was significantly lower among nurses who worked in settings where there were more than 15 patients per RN per shift. According to Korean medical laws, the daily patient census per RN is 2.5, which converts to an estimated patient-per-RN rate of approximately 15 per shift. The average estimated patient-per-RN rate per shift in Korean general hospitals from 1996 to 2013 was 16.3 to 21.8, which exceeded the minimum standards required by law [33]. Failure to obtain an adequate number of nurses leads to increased job burden, insufficient nursing time, and decreased job performance ability [34], and an increased rate of infections [17,34]. Accordingly, sufficient numbers of appropriately qualified nurses or optimal nurse-to-patient ratio should be considered to increase the rate of adherence to standard precautions for patient safety.

This study demonstrated that there was a significant positive correlation between nurses’ standard precaution adherence and their perceptions of patient safety management. More importantly, the multiple linear regression results revealed that nurses’ perceptions of patient safety management were only significantly associated with adherence to standard precautions after adjusting for other covariates. As nurses’ perceptions of patient safety management may affect nursing activities [14,26,35], raising nurses’ awareness about patient safety management must be prioritized to increase adherence to standard precautions. Particularly, practical guidelines and continuous training to increase nurses’ positive awareness, interest, and willingness to participate in patient safety management can contribute to improvements in practicing standard precautions [36]. Despite no observed significant relationship between nurse-to-patient ratio and adherence to standard precautions in hierarchical analysis, our correlation matrix showed that nurse-to-patient ratio was weakly associated with adherence to standard precautions. According to a recent review [37], low adherence to standard precautions may be caused by inappropriate work conditions (excessive workload and reduced teams). Thus, intervention studies are needed in future to improve work conditions such as appropriate staff sizing.

This study had some limitations. Due to the cross-sectional research design and focus on a single hospital, there was a lack of causality. Additionally, the convenience sample makes it difficult for generalizing the findings. Therefore, the generalizability of these findings should be investigated using a larger cohort sample as well as in multiple health care settings. This study also used a self-report questionnaire; thus, there might have been discrepancies between the nurses’ self-assessment of their adherence to standard precautions and objective assessments.

## 5. Conclusions

This study found that the overall adherence rate to standard precautions among hospital nurses was suboptimal. Our main finding highlights that nurses’ perceptions of patient safety management were associated with adherence to standard precautions among hospital nurses. This study suggests that supportive, specialized training in wearing personal protective equipment has to be executed well, considering nurses’ work environments—including the total number of nurses working together and the nurse-to-patient ratio. Further, nurse educators should develop an integrated undergraduate curriculum regarding patient safety management linked to clinical nurse education to help facilitate a smooth transition to clinical practice. Future studies are needed to investigate the causal relationships between nurses’ work environments including patient safety culture and compliance with infection control practices in clinical settings.

## Figures and Tables

**Table 1:** Socio-demographics and work-related characteristics of participants. *N =* 329.

Variable	Category	*N*(%)	Mean ± SD
Age (years)	22–25	77 (23.4)	29.48 ± 4.50
	26–30	127 (38.6)	
	≥31	125 (38.0)	
Marital status	Married/Partnered	87 (26.4)	
	Single	242 (73.6)	
Educational level	College (3 years)	51 (15.5)	
	University (4 years)	243 (73.9)	
	Graduate school	35 (10.6)	
Total years in practice	<5	155 (47.1)	6.74 ± 4.72
	5–9	81 (24.6)	
	10–14	72 (21.9)	
	≥15	21 (6.4)	
Years in practice in current job	<5	202 (61.4)	4.84 ± 3.80
	5–9	82 (24.9)	
	≥10	45 (13.7)	
Current position	Staff nurse	273 (83.0)	
	Charge nurse	56 (17.0)	
Area of practice	Medical ward	103 (31.3)	
	Surgical ward	82 (24.9)	
	Intensive care unit	65 (19.8)	
	Emergency department	20 (6.1)	
	Others	59 (17.9)	
Total number of nurses working together (including nursing assistants)	<20	43 (13.0)	31.61 ± 13.10
	20–39	218 (66.3)	
	40–60	68 (20.7)	
Nurse-to-patient ratio	<5	69 (20.9)	10.25 ± 4.63
	5–9	64 (19.5)	
	10–14	116 (35.3)	
	≥15	80 (24.3)	
Working hours per week (including overtimes)	40	113 (34.3)	44.57 ± 4.06
	41–47	115 (35)	
	≥48	101 (30.7)	
Prior experience in patient safety education	Yes	320 (97.3)	
	No	9 (2.7)	
Prior experience in infection control education	Yes	321 (97.6)	
	No	8 (2.4)	
Perception of patient safety management			3.89 ± 0.46

SD, standard deviation; others, neonatal ward, delivery unit, psychiatric ward.

**Table 2:** Adherence rate on standard precautions among hospital nurses. *N =* 329.

Rank	Item	Adherence Rate	
		*N*	(%)
1	I put used sharp articles into sharps box	312	94.8
2	My mouth and nose are covered when I wear a mask	243	73.9
3	I decontaminate my hands immediately after removal of gloves	243	73.9
4	I wash my hands between patient contacts	227	69.0
5	I clean up spillage of blood or other body fluid immediately with disinfectants	226	68.7
16	I take a shower in case of extensive splashing even after I have put on personal protective equipment	128	38.9
17	* I only use water for hand washing	117	35.6
18	* The sharps box is only disposed when it is full	110	33.4
19	I wear a surgical mask alone or in combination with goggles, face shield, and apron whenever there is a possibility of a splash or splatter	106	32.2
20	I wear a gown or apron when exposed to blood, body fluids, or any patient excretions	59	17.9
Mean ± SD 10.71 ± 4.39 (Average adherence rate: 53.5%)			

Scale items were arranged from the highest to lowest adherence rate. * Reverse scored items.

**Table 3:** Differences in adherence to standard precautions by socio-demographics and work-related characteristics of participants. *N =* 329.

Variables	Category	Mean ± SD	*t* or F	*p*
Age (years)	22–25	10.13 ± 4.36	1.626	0.198
	26–30	10.54 ± 4.39		
	≥31	11.22 ± 4.38		
Marital status	Married/Partnered	10.86 ± 4.48	−0.388	0.698
	Single	10.65 ± 4.36		
Educational level *	College (three years)	11.08 ± 4.11	5.301	0.357
	University (four years)	10.52 ± 4.31		
	Graduate school	11.43 ± 5.29		
Total years in practice	<5	10.44 ± 4.30	0.369	0.775
	5–9	10.88 ± 4.62		
	10–14	11.00 ± 4.27		
	≥15	11.00 ± 4.80		
Years in practice in current job	<5	10.57 ± 4.41	0.261	0.771
	5–9	10.88 ± 4.39		
	≥10	11.00 ± 4.38		
Current position	Nurse	10.64 ± 4.36	−0.584	0.559
	Charge nurse	11.02 ± 4.56		
Area of practice	Medical ward	10.71 ± 4.59	1.489	0.205
	Surgical ward	10.16 ± 4.71		
	Intensive care unit	10.89 ± 3.90		
	Emergency department	9.45 ± 4.31		
	Others	11.68 ± 4.02		
Total number of nurses † (including nursing assistants)	<20 ^a^	12.19 ± 4.47	3.256	0.040
	20–39 ^b^	10.35 ± 4.31	a > b	
	40–60 ^c^	10.90 ± 4.44		
Nurse-to-patient ratio †	<5 ^a^	10.83 ± 4.03	2.693	0.046
	5–9 ^b^	11.69 ± 4.52	b > d	
	10–14 ^c^	10.82 ± 4.21		
	≥15 ^d^	9.65 ± 4.69		
Working hours per week (including overtimes)	<40	10.66 ± 4.52	0.060	0.942
	40–47	10.82 ± 4.20		
	≥48	10.63 ± 4.55		
Prior experience in patient safety education	Yes	10.76 ± 4.41	1.415	0.158
	No	8.67 ± 3.43		
Prior experience in infection control education	Yes	10.72 ± 4.42	0.459	0.646
	No	10.00 ± 3.03		

* H value of Kruskal-Wallis; † significant difference between groups in Bonferroni post hoc test; SD, standard deviation.

**Table 4:** Correlation between measured variables. *N* = 329.

Variables	Age	Marital Status	Total Years in Practice	Years in Practice in Current Job	Current Position	Total Number of Nurses	Nurse-to- Patient Ratio	Perception of Patient Safety Management	Adherence to Standard Precautions
	*r/rho/tau* (*p*)	*r/rho/tau* (*p*)	*r/rho/tau* (*p*)	*r/rho/tau* (*p*)	*r/rho/tau* (*p*)	*r/rho* (*p*)	*r/rho* (*p*)	*r* (*p*)	*r* (*p*)
Age	1								
Marital status	0.563 (<0.001)	1							
Total years in practice	0.921 (<0.001)	0.542 (<0.001)	1						
Years in practice in current job	0.596 (<0.001)	0.320 (<0.001)	0.649 (<0.001)	1					
Current position	0.562 (<0.001)	0.362 (<0.001)	0.576 (<0.001)	0.434 (<0.001)	1				
Total number of nurses	−0.136 (0.013)	−0.041 (0.460)	−0.127 (0.021)	−0.049 (0.372)	0.055 (0.316)	1			
Nurse-to-patient ratio	−0.065 (0.238)	−0.054 (0.329)	−0.012 (0.825)	0.004 (0.946)	−0.077 (0.166)	−0.327 (<0.001)	1		
Perception of patient safety management	0.201 (<0.001)	0.171 (0.002)	0.213 (<0.001)	0.086 (0.119)	0.207 (<0.001)	−0.057 (0.304)	−0.051 (0.358)	1	
Adherence to standard precautions	0.105 (0.056)	0.049 (0.374)	0.083 (0.135)	0.617 (0.329)	0.041 (0.460)	−0.042 (0.451)	−0.123 (0.026)	0.413 (<0.001)	1

**Table 5:** Multiple linear regression analysis predicting adherence to standard precautions. *N =* 329.

Variables	Step 1			Step 2		
	*β*	*t (p)*	95% CI	*β*	*t* (*p*)	95% CI
Age (years)	0.096	1.096 (0.274)	−0.074 to 0.262	0.078	0.965 (0.335)	−0.079 to 0.230
Marital status (reference = single)	–0.013	−0.202 (0.840)	−1.371 to 1.115	–0.040	–0.654 (0.514)	−1.520 to 0.762
Years in practice in current job	0.008	0.115 (0.908)	−0.013 to 0.015	0.005	0.071 (0.943)	−0.011 to 0.013
Current position (reference = staff nurse)	–0.030	−0.407 (0.695)	−2.058 to 1.353	–0.090	−1.306 (0.192)	−2.167 to 0.529
Total number of nurses	–0.116	−1.736 (0.084)	−0.083 to 0.005	–0.061	−0.988 (0.324)	−0.061 to 0.020
Nurse-to-patient ratio	−0.135	−2.055 (0.040)	−0.250 to −0.006	–0.093	−1.554 (0.121)	−0.201 to 0.024
Perception of patient safety management				0.412	7.894 (<0.001)	0.140 to 0.234
*Adjusted R*^2^, F (*p*), △*R*^2^	0.007, 1.314 (0.243)			0.166, 10.302 (<0.001), 0.159		

CI, confidence interval.

## References

[1] Haile T.G., Engeda E.H., Abdo A.A. (2017). Compliance with standard precautions and associated factors among healthcare workers in Gondar University Comprehensive Specialized Hospital, Northwest Ethiopia. J. Environ. Public Health.

[2] Hessels A.J., Larson E.L. (2016). Relationship between patient safety climate and standard precaution adherence: A systematic review of the literature. J. Hosp. Infect..

[3] Rosinki J., Rozanska A., Jarynowski A., Wojkowska-Mach J., Polish Society of Hospital Infection Team (2019). Factors shaping attitude of medical staff towards acceptance of the standard precautions. Int. J. Environ. Res. Public Health.

[4] Rozenbojm M.D., Nichol K., Spielmann S., Holness D.L. (2015). Hospital unit safety climate: Relationship with nurses’ adherence to recommended use of facial protective equipment. Am. J. Infect. Control.

[5] Korea Centers for Disease Control and Prevention (2017). Guidelines for Prevention and Control of Healthcare Associated Infection.

[6] Ki M. (2015). 2015 MERS outbreak in Korea: Hospital-to-hospital transmission. Epidemiol. Health.

[7] Kim K.W., Jang S.-N. (2018). Who comes to the emergency room with an infection from a long-term care hospital? A retrospective study based on a medical record review. Asian Nurs. Res..

[8] World Health Organization Patient Safety. https://www.who.int/patientsafety/en/.

[9] Fernandes Agreli H., Murphy M., Creedon S., Bhuachalla C.N., O’Brien D., Gould D., Savage E., Barry F., Drennan J., Smiddy M.P. (2019). Patient involvement in the implementation of infection prevention and control guidelines and associated interventions: A scoping review. BMJ Open.

[10] Siegel J.D., Rhinehart E., Jackson M., Chiarello L. (2007). Guideline for Isolation Precautions: Preventing Transmission of Infectious Agents in Healthcare Settings. https://www.cdc.gov/niosh/docket/archive/pdfs/NIOSH-219/0219-010107-siegel.pdf.

[11] Kim J.E., Shin K.A., Shin M.K., Shin J.J., Lee H.H. (2018). Challenges in Korea hospital accreditation: Focused on post-accreditation management system. Qual. Improv. Health Care.

[12] Luo Y., He G.-P., Zhou J.-W., Luo Y. (2010). Factors impacting compliance with standard precautions in nursing, China. Int. J. Infect. Dis..

[13] Donati D., Biagioli V., Cianfrocca C., De Marinis M.G., Tartaglini D. (2019). Compliance with standard precautions among clinical nurses: Validity and reliability of the Italian version of the Compliance with Standard Precautions Scale (CSPS-It). Int. J. Environ. Res. Public Health.

[14] Hessels A.J., Genovese-Schek V., Agarwal M., Wurmser T., Larson E.L. (2016). Relationship between patient safety climate and adherence to standard precautions. Am. J. Infect. Control.

[15] Suh Y.H., Oh H.Y. (2010). Knowledge, perception, safety climate, and compliance with hospital infection standard precautions among hospital nurses. J. Korean Clin. Nurs. Res..

[16] Tae S.H., Hwang E.H. (2012). Nurses’ clinical competence and its relationship with perception of and compliance with standard precautions. Korean J. Health Promot..

[17] Shang J., Stone P., Larson E. (2015). Studies on nurse staffing and health care–associated infection: Methodologic challenges and potential solutions. Am. J. Infect. Control.

[18] Colet P.C., Cruz J.P., Alotaibi K.A., Colet M.K., Islam S.M. (2017). Compliance with standard precautions among baccalaureate nursing students in a Saudi university: A self-report study. J. Infect. Public Health.

[19] Nofal M., Subih M., Al-Kalaldeh M. (2017). Factors influencing compliance to the infection control precautions among nurses and physicians in Jordan: A cross-sectional study. J. Infect. Prev..

[20] Kim I.-S., Park M., Park M.-Y., Yoo H., Choi J. (2013). Factors affecting the perception of importance and practice of patient safety management among hospital employees in Korea. Asian Nurs. Res..

[21] Hossan M., Sheuli M.S., Nesa M., Lee T.W. (2018). Nurses’ perception on patient safety in hospital setting. Int. J. Sci. Eng. Res..

[22] Park M.J., Kim I.S., Ham Y.L. (2013). Development of a perception of importance on patient safety management scale (PI-PSM) for hospital employee. J. Korean Cont. Assoc..

[23] Faul F., Erdfelder E., Lang A.-G., Buchner A. (2007). G* Power 3: A flexible statistical power analysis program for the social, behavioral, and biomedical sciences. Behav. Res. Methods.

[24] Thiese M.S. (2014). Observational and interventional study design types: An overview. Biochem. Med..

[25] Lam S.C. (2011). Universal to standard precautions in disease prevention: Preliminary development of compliance scale for clinical nursing. Int. J. Nurs. Stud..

[26] Alingh C.W., van Wijngaarden J.D., van de Voorde K., Paauwe J., Huijsman R. (2019). Speaking up about patient safety concerns: The influence of safety management approaches and climate on nurses’ willingness to speak up. BMJ Qual. Saf..

[27] Cruz J.P., Colet P.C., Al-otaibi J.H., Soriano S.S., Cacho G.M., Cruz C.P. (2016). Validity and reliability assessment of the compliance with standard precautions scale Arabic version in Saudi nursing students. J. Infect. Public Health.

[28] Pereira F.M.V., Lam S.C., Chan J.H.M., Malaguti-Toffano S.E., Gir E. (2015). Difference in compliance with standard precautions by nursing staff in Brazil versus Hong Kong. Am. J. Infect. Control.

[29] Kim K.M., Oh H. (2015). Clinical experiences as related to standard precautions compliance among nursing students: A focus group interview based on the theory of planned behavior. Asian Nurs. Res..

[30] Shin H.Y., Kim K.H., Kim K.S. (2011). Study on pediatric nurses’ attitudes and compliance with hospital infection standard precautions. Child Health Nurs. Res..

[31] Quan M., Wang X., Wu H., Yuan X., Lei D., Jiang Z., Li L. (2015). Influencing factors on use of standard precautions against occupational exposures to blood and body fluids among nurses in China. Int. J. Clin. Exp. Med..

[32] Thukral A., Lockyer J., Bucher S.L., Berkelhamer S., Bose C., Deorari A., Esamai F., Faremo S., Keenan W.J., McMillan D. (2015). Evaluation of an educational program for essential newborn care in resource-limited settings: Essential care for every baby. BMC Pediatr..

[33] Cho S.-H., Lee J.-Y., June K.-J., Hong K.J., Kim Y. (2016). Nurse staffing levels and proportion of hospitals and clinics meeting the legal standard for nurse staffing for 1996~2013. J. Korean Acad. Nurs. Adm..

[34] Kim M.-G. (2017). The impact of nurse staffing level on in-hospital death and infection in cancer patients who received surgery. J. Korean Acad. Ind. Coop. Soc..

[35] Jimmieson N.L., Tucker M.K., White K.M., Liao J., Campbell M., Brain D., Page K., Barnett A.G., Graves N. (2016). The role of time pressure and different psychological safety climate referents in the prediction of nurses’ hand hygiene compliance. Saf. Sci..

[36] Kim L., Lyder C.H., McNeese-Smith D., Leach L.S., Needleman J. (2015). Defining attributes of patient safety through a concept analysis. J. Adv. Nurs..

[37] Porto J.S., Palucci Marziale M.H. (2016). Reasons and consequences of low adherence to standard precautions by the nursing team. Rev. Gauch. Enferm..

